# Income, occupation and education: Are they related to smoking behaviors in China?

**DOI:** 10.1371/journal.pone.0192571

**Published:** 2018-02-08

**Authors:** Qing Wang, Jay J. Shen, Michelle Sotero, Casey A. Li, Zhiyuan Hou

**Affiliations:** 1 School of Business, Dalian University of Technology, Panjin, Liaoning, China; 2 Department of Health Care Administration and Policy, School of Community Health Sciences, University of Nevada Las Vegas, Las Vegas, Nevada, United States of America; 3 Green Valley High School, Henderson, Nevada, United States of America; 4 Department of Social Medicine, School of Public Health, Fudan University, Shanghai, China; 5 National Key Laboratory of Health Technology Assessment (Health and Family Planning Commission), Fudan University, Shanghai, China; 6 Collaborative Innovation Center of Social Risks Governance in Health, Fudan University, Shanghai, China; CUNY, UNITED STATES

## Abstract

**Background:**

The association between socioeconomic status (SES) and smoking behaviors may differ across countries. This study aimed to estimate the association between socioeconomic status (income, occupation and education) and multiple measures of smoking behaviors among the Chinese elderly population.

**Methods:**

Using data from the China Health and Retirement Longitudinal Study in 2013, we examined the relationship between socioeconomic status and smoking behaviors through multivariate regression analysis. Sample selection models were applied to correct for sample selection bias. Smoking behaviors were measured by four indicators: smoking status, cigarette consumption, health risks related to smoking, and smoking dependence. Analyses were stratified by gender and urban-rural residence.

**Results:**

Among Chinese people aged 45 years or older, smokers accounted for 40% of the population in 2013, smoking 19 cigarettes per day. It was also found that 79% of smokers were at an increased health risk. Overall, although the influence of income on smoking behaviors was small and even insignificant, occupation and education levels were significantly associated with smoking behaviors. Managers or professionals were more likely to smoke, however there was no significant relationship with smoking dependence. Individuals with higher educational attainment were less likely to be associated with smoking behaviors. In addition, gender and urban-rural differences existed in the relationship between SES and smoking behaviors.

**Conclusions:**

Smoking disparities among diverse levels of socioeconomic status existed but varied greatly by SES indicators and population characteristics. Tobacco control policies in China should be increasingly focused on populations with low socioeconomic status in order to break the link between socioeconomic disadvantage and smoking behaviors. Further actions should mitigate inequalities in education, improve the social culture of cigarette use, and tailor interventions based on characteristics of the population.

## Introduction

Cigarette smoking is a widespread unhealthy behaviors and a leading cause for premature death [1]. Globally, about 20% of adults smoke cigarettes, resulting in approximately 100 million deaths over the course of the 20th century [2]. Socioeconomic status (SES) has been considered an important determinant of smoking behaviors [3]. According to the theory of diffusion of innovation, four stages of the smoking epidemic have been described [4, 5]. In the first stage, smoking pervades the higher socioeconomic groups (innovators). During the second stage, smoking spreads to the rest of the population, including the lower socioeconomic groups (laggards). The third stage is characterized by the start of cessation in higher socioeconomic groups, male dominance, and a rise in female smoking. Finally, in the fourth stage, smoking declines among higher socioeconomic groups, but remains high among lower SES [6]. Thus, effects of SES on smoking behaviors may differ across countries with different socioeconomic development levels. Empirical studies seem to support the theory hypothesis to some extent. In most high-income countries (e.g. the USA, Canadian, Japan and Korea), the prevalence of smoking was inversely related to social position [7–15]. In contrast, a positive association between SES and tobacco use was found in some developing countries, such as India, Thailand and Malaysia [16–23].

SES has different measures, including but not limited to education, income and occupation. However, most studies focus on smoking disparities by education rather than other measures. Although education is an important socioeconomic indicator, studies suggest other SES indicators (e.g. occupation and income) may independently influence smoking [19,22]. Each of these indicators may influence smoking behaviors through different mechanisms. For example, education may reflect a person’s knowledge and ability to make health-conscious decisions, including smoking behaviors [24]. Occupation, a measure of one’s position in the socioeconomic hierarchy, is more closely connected with working conditions than other socioeconomic indicators [7]. Household income, indicating one’s possession of material resources, may present access to tobacco and tobacco control interventions. Psychologically speaking, it has been found that people may smoke to cope with stress induced by socioeconomic circumstances [8].

In addition, the relative importance of different SES measures may change over the course of an individual’s life. Education, which is generally determined during adolescence and early adulthood, might be a strong predictor of exposure to tobacco and smoking behaviors. In contrast, the relevance of income and occupation may increase for older adults, affecting cigarette consumption levels and smoking cessation [9]. Recent studies conducted in the European Union, Estonia, Thailand, and Malaysia found that education and income affected smoking behaviors differentially [11,19].

The various measures of smoking behaviors may play a role on the unique relationship with SES. In addition to the prevalence rates of smoking, several studies employed information on smoking initiation, cessation rates, and consumption levels to investigate smoking disparities by SES [7]. The effects of SES on different smoking measures were found heterogeneous [20, 21]. Among well-educated Canadian citizens, smoking ratios decreased while cessation ratios increased by education [25]. In Thailand, a higher level of education was found to be strongly associated with smoking prevalence, but not associated with cigarette consumption. In Malaysia, however, higher income was strongly associated with having self-efficacy to quit but not associated with the other smoking measures [21].

Given the variation in the association of SES and smoking behaviors across countries, it is particularly interesting to examine the issue in China contexts. China, the leading country in cigarette production and consumption, produced and consumed more than 30% of the world’s total cigarettes in 2014 [26, 27]. Tobacco control policies in China are usually examined for the national population as a whole and without a specific focus on socioeconomic inequalities. This may come from the lack of comprehensive understanding of the SES differences in relation to smoking behaviors [7]. Therefore, it is necessary to identify high-risk SES populations for smoking behaviors and track the patterns of tobacco consumption by SES. This information may be helpful to tailor tobacco control policies in China.

In addition, the association between SES and smoking behaviors may differ by gender and rural-urban areas. In the international literature, patterns of smoking disparities in SES were found to differ by gender. Among European Union countries, income was related to smoking for males but not for females.^7^ In regard to smoking initiation in France, no educational gradient appeared in males, but there was a positive gradient in women [18]. In South Korea, SES was inversely associated with smoking among both genders. In China, smoking behaviors were predominantly driven by males. According to the 2013 wave of China Health and Retirement Longitudinal Study (CHARLS), 74% of males were smokers, whereas only 8% of females were smokers [1]. China was found to be one of the countries with the highest male-to-female ratio of smoking prevalence. The CHARLS data also showed that urban residents reported higher SES than rural residents. Therefore, it is also meaningful to examine the association between SES and smoking behaviors by rural-urban areas in China.

According to our knowledge, no study has utilized various measures to compare multiple SES differentials in smoking behaviors with nationally representative data in China [28–34]. Therefore, using the CHARLS national data, this study aims to estimate the relationship between SES (including the income, occupation, and educational attainment) and smoking behaviors among the Chinese population aged 45 and above. We further stratified our analysis to examine the association of SES and smoking by gender and rural-urban areas. This may be the first study to evaluate the effects of SES on multiple measures of smoking behaviors among the under-studied elderly population in China.

## Methods

### Study design and data

This study used data from the 2013 wave of CHARLS, which is publicly available on the following website: http://charls.pku.edu.cn/en. CHARLS is a nationally representative sample of Chinese residents aged 45 and above. The cut-off age of CHARLS was chosen because 45 is the minimum retirement age (the minimum age for receiving pension) in China [35]. For the purposes of our study, the higher smoking prevalence among this sample made the population attractive [36]. It covers approximately 10,000 households in 150 counties/districts (a total of 450 villages/resident communities) and adopts the multi-stage stratified probability proportional to its size sampling technique. CHARLS is similar to the Health and Retirement Study in the United States, the English Longitudinal Study of Aging in the United Kingdom, and the Survey of Health, Aging and Retirement in Europe. The CHARLS questionnaire includes the following modules: demographics, family structure, health status, functioning, biomarkers, income, consumption, assets (individual and household), and community-level information. Ethical consent was approved by the Institutional Review Board of Peking University, and informed written consents were obtained from the participants. After excluding the observations with missing values, 17,495 individual observations were available for statistical analysis.

### Measure of smoking behaviors

Smoking behaviors were measured by four indicators: smoking status, cigarette consumption, health risks related to smoking, and smoking dependence. Each measure depicted different phases of smoking behaviors. By using these measures together, we were able to obtain a complete picture of overall smoking tendencies among respondents. Smoking status was classified into two categories: smoker, and non-smoker. Smoker was classified as respondents who have ever smoked or currently smoke. Cigarette consumption was defined as the number of cigarettes an individual smoked per day, which was based on the responses to the following question from the CHARLS questionnaire: “How many cigarettes do you smoke each day now?” Moreover, log transformation of cigarette consumption was used for the analysis to minimize skewness [37].

Health risks related to smoking were measured by smoking index, combining measures of smoking intensity and duration. The smoking index was calculated using the number of cigarettes smoked daily multiplied by the years of smoking. For instance, if an individual smoked for 20 years and, on average, smoked a pack of cigarettes per day, his/her smoking index was 20×20 = 400. A person whose smoking index is equal to or greater than 400 is regarded as a heavy smoker and considered to have a high risk for lung cancer [38]. Therefore, a dummy variable was used to indicate high-risk smokers or not. The variable takes the value of 1 for high-risk smokers and 0 for non-high-risk smokers.

In addition, some smokers try to quit smoking, but fail repeatedly. Nicotine dependence is a great barrier to smoking cessation since smoking prevents and reduces nicotine withdrawal symptoms [39–42]. Individuals addicted to smoking were identified as the most vulnerable group to health risks associated with smoking. Thus, the last indicator was smoking dependence, which was measured by the Heaviness of Smoking Index (HSI). In the CHARLS questionnaire, the only measure of nicotine dependence is the HSI. The HSI score is calculated based on two items: the number of cigarettes smoked per day and the time of the first cigarette after waking up, both taking values from 0 to 3. The number of cigarettes smoked per day was recoded into an ordinal variable from 0 to 3 representing the level of smoking (0 = smoked 0–9 cigarettes per day; 1 = smoked 10–19 cigarettes per day; 2 = smoked 20–29 cigarettes per day, 3 = smoked more than 30 cigarettes per day). The time of the first cigarette after waking up was classified into 4 groups with 0 = more than 1 hour, 1 = within 31–60 minutes, 2 = with 6–30 minutes and 3 = within 5 minutes. These two items are then summed and as a result, the value of the HSI ranges from 0 to 6 [43, 44]. Smoking dependence was then classified into three ordered categories: 0, 1–3, and 4–6.

In the CHARLS survey, all smoking data was self-reported. Validation studies indicate that self-reported measures of smoking behaviors are broadly reliable for adult and non-pregnant populations, with no systematic socioeconomic biases in under-reporting [45, 46].

### Measure of SES

Our independent variables of interest consisted of three SES measures: income, occupation, and educational attainment. They are the most commonly used SES measures in health and smoking behavioral research [9, 47].

Household income per capita was used to measure income in our study and was captured by several income questions in CHARLS [48]. The income questions pertained to labor earnings such as wages and salaries or self-employment income, non-labor income including interest, dividends, or rental income, private transfer income such as alimony or workers’ compensation and public transfer income such as unemployment insurance, welfare, social security. Irregular income, such from stock options and capital gains, was not considered. Family size was defined as a census subfamily, which includes all related individuals in a household. Accordingly, the income was adjusted for family size by dividing it. Subsequently, household income per capita was ranked and divided into three quintiles (high, middle and low), with the low group as the reference group.

Occupation categories were coded from self-reported job descriptions. According to Erikson and Goldthorpe and Portocarero Class Categories, occupation was compressed to the following five categories for this study: managers and professionals, self-employed, agricultural workers, manual workers, and unemployed, with unemployed as the reference group [49, 50].

Education was measured by a question referencing completed general or vocational education. Educational attainment was attributed at five levels: no formal education, no formal education but can read and write, elementary school, junior high school, and high school and above. Four dummy variables were created for educational attainment, with no formal education serving as the reference group.

### Statistical analysis

We first conducted the descriptive analysis of the sample characteristics totally and separately for those over the age of 45 by gender and urban-rural residence. To control for individual demographic characteristics, multivariate regressions were then used to identify the association between SES measures and smoking behaviors. According to CHARLS 2013, the large variation in smoking behaviors was revealed by gender and SES differences by urban-rural residence. As a result, regression models were fitted separately by gender and urban-rural residence.

A nonrandom sample selection may occur because cigarette consumption, health risks related to smoking, and smoking dependence may hinge on the decision to smoke (smoking status). To address this issue, the sample selection model (SSM), a two-stage procedure that addresses sample selection bias in the regression analysis, can be applied. The SSM estimates the parameters in two stages with a selection equation and an outcome equation. The selection equation predicts the influence of each independent variable on prevalence of the smoker, controlling for individual demographic and SES factors and their father’s education. The father’s education was classified into no formal education, no formal education but can read and write, elementary school, and junior high school and above, with no formal education serving as the reference group. The father’s education was found to be a determinant of smoking status but did not significantly affect cigarette consumption levels [51]. Demographic variables included age, gender, marital status, and urban or rural residence.

The outcome equation predicts cigarette consumption, health risks related to smoking, and smoking dependence, conditional on the selection equation. When the error terms from these two equations are significantly correlated, standard regression techniques applied to the outcome equation alone can yield biased results. As a result, the issue of selection bias needs to be addressed [52].

To check the sample selection bias, the correlation in the error terms and the significance of the t-ratio associated with the inverse Mills ratio (IMR) were tested. Based on the IMR test result, the association of SES and cigarette consumption would be affected by the sample selection bias for males (IMR: 3.34; P: 0.014). Hence, the SSM was applied to evaluate the association of SES and cigarette consumption for the male sample. However, sample selection bias was not observed to influence the relationship between SES and other smoking measures. Therefore, the ordinary least squares (OLS) and Logit or ordered Logit regressions were applied for the smoker subsample to estimate the relationship between SES and health risks related to smoking, and smoking dependence respectively. Odds Ratios (OR) with 95% Confidence Intervals (CI) were reported for Logit / Ordered Logit regressions. Coefficients with Standard Error (SE) were reported for OLS/SSM regressions. All statistical analyses were performed using STATA 14.0.

## Results

### Characteristics of the survey respondents

Table 1 presents the descriptive statistics of the CHARLS 2013 sample. About 40% of the respondents over the age of 45 identified themselves as smokers. The average number of cigarettes smoked per day was 19, with a HSI score of 2.57. Additionally, 79% of them were considered to be at high health risk as measured by the smoking index.

**Table 1:** Characteristics of the survey respondents, CHARLS 2013 (%).

	Total	Male	Female	Urban	Rural
	n = 17,495	n = 8,389	n = 9,106	n = 7,042	n = 10,453
**Smoking behaviors**					
Smoking status					
Smoker	39.66	73.84	8.17	37.42	41.17
Non-smoker	60.34	26.16	91.83	62.58	58.83
Cigarette consumption for smokers (Mean±SD)	19.00±12.57	19.61±11.64	13.92±12.64	18.47±12.49	19.32±12.62
Smoking Initiation for smokers (Age, Mean±SD)	22.77±8.92	22.30±8.09	26.68±13.29	22.52±8.92	22.92±8.91
High health risks related to smoking for smokers	78.51	79.84	67.43	74.41	81.02
Smoking dependence for smokers (Mean±SD)	2.57±1.96	2.57±1.94	2.56±2.12	2.29±1.89	2.74±1.98
**Socio-economic status (SES)**					
**Income**					
Household income per capita (CNY, Mean±SD) ^a^	7631.42±11640.90	7735.60±11935.93	7536.89±11362.3	11285.12±14975.88	5170.14±7796.84
**Employment Status**					
Unemployed	17.93	11.28	24.06	16.74	18.73
Manual workers	22.86	23.05	22.65	37.45	13.03
Agricultural workers	36.73	35.24	38.10	17.88	49.44
Self-employed	7.79	9.55	6.17	9.44	6.68
Managers and professionals	14.69	20.88	8.99	18.49	12.13
**Education**					
No formal education	27.17	12.87	40.35	17.20	33.89
No formal education but can read/write	17.81	18.15	17.50	14.98	19.72
Elementary school	21.59	25.99	17.53	20.51	22.32
Primary school	23.18	29.78	17.10	29.88	18.67
High school and above	10.25	13.21	7.52	17.44	5.41
**Demographic characteristics**					
Male	47.95	100	0	46.71	48.79
Age (Mean±SD)	59.03±10.13	59.55±9.73	58.56±10.47	58.93±10.26	59.10±10.04
Unmarried	12.71	9.27	15.88	12.31	12.98
Urban residence	40.25	39.21	43.21	100	0
**Father’s education**					
No formal education	55.58	59.94	62.64	47.25	64.60
No formal education but can read/write	11.19	10.53	11.63	12.69	9.58
Elementary school	23.65	21.17	25.30	27.07	19.94
Primary school and above	9.58	8.35	10.4	12.99	5.89

^a^ 1 USD = 6.88 CNY, 2017-01-31. Smokers include current and ever smokers. SD: Standard deviations.

Overall, 48% of the respondents were male, and the average age was 59 years. The household income per year per capita was 7,631 CNY ($1,108 USD). About 15% of the respondents were managers and professionals, 8% self-employed, 37% agricultural workers, and 23% manual workers. Nearly half of those surveyed had no formal education and only 10% had high school education or above.

It was found that 74% of males and 8% of females were smokers. On average, males and females smoked 20 and 14 cigarettes daily respectively. There were 80% of males and 67% of females with high health risks related to smoking. Compared to females, males reported higher SES measured by a higher proportion of managers and professionals, higher household income per capita, and educational attainment.

Smoking behaviors and SES were also different between urban and rural residents. As expected, urban residents had higher SES than rural residents. In comparison with urban residents, rural residents had higher smoking prevalence (41% vs 37%), higher health risks (81% vs 74%), and higher smoking dependence (2.74 vs 2.29).

### Association between SES and smoking behaviors

Table 2 shows the association of SES and smoking status controlling for all other covariates by Logit models among the middle aged and elderly Chinese population. Higher income and being managers or professionals were positively associated with being a smoker for males, whereas no significant association was found for females. Income and occupation had little influence on smoking status both for urban and rural residents. Compared with respondents with no formal education, those with no formal education but can read/write significantly increased the smoking probability. Those with high school education or above decreased the smoking probability regardless of their gender or residence.

**Table 2:** Association between SES and smoker using Logit model (non-smoker as reference): OR with 95% CI.

VARIABLES	Male	Female	Urban	Rural
	OR	OR	OR	OR
	(95%CI)	(95%CI)	(95%CI)	(95%CI)
**Income (low level as reference)**				
Middle level	1.13	1.00	1.00	1.11
	(0.98–1.30)	(0.81–1.23)	(0.80–1.26)	(0.97–1.28)
High level	1.17**	0.88	1.09	1.02
	(1.00–1.36)	(0.69–1.11)	(0.87–1.35)	(0.87–1.20)
**Occupation (the unemployed as reference)**				
Manual workers	1.15	0.84	0.91	0.86
	(0.92–1.44)	(0.64–1.11)	(0.69–1.19)	(0.67–1.10)
Agricultural workers	1.42***	0.90	1.04	1.11
	(1.16–1.74)	(0.71–1.13)	(0.78–1.39)	(0.92–1.35)
Self-employed	1.18	0.63*	0.84	1.00
	(0.91–1.52)	(0.40–1.02)	(0.60–1.18)	(0.76–1.32)
Managers and professionals	1.46***	0.66*	1.00	1.27
	(1.15–1.85)	(0.42–1.03)	(0.73–1.37)	(0.99–1.64)
**Education (no formal education as reference)**				
No formal education but can read/write	1.37***	1.23*	1.20	1.25**
	(1.10–1.70)	(0.97–1.56)	(0.89–1.64)	(1.03–1.51)
Elementary school	0.98	1.09	0.98	0.92
	(0.80–1.19)	(0.85–1.41)	(0.73–1.32)	(0.76–1.10)
Primary school	0.92	0.76	0.88	0.87
	(0.75–1.14)	(0.55–1.07)	(0.65–1.19)	(0.71–1.07)
High school and above	0.75**	0.62*	0.68**	0.79*
	(0.59–0.95)	(0.38–1.01)	(0.49–0.94)	(0.61–1.02)
N	8,389	9,106	7,042	10,453

Note: Demographic characteristics and father’s education were controlled.

Significance level:

*** p<0.01,

** p<0.05,

* p<0.10.

OR: odd ratio. CI: confidence interval.

Table 3 shows the association of SES and cigarette consumption by SSM or OLS models among the middle aged and elderly Chinese population. The results showed that income was insignificantly related to cigarette consumption. Compared with the unemployed, managers and professionals consumed more cigarettes by 0.09 and 0.17 in urban and rural areas respectively. Those with a high school education or above significantly decreased cigarette consumption for females and urban respondents by 0.05 and 0.23 respectively.

**Table 3:** Association between SES and cigarette consumption using the sample selection model /OLS: Coefficient with standard error.

	Male	Female	Urban	Rural
**Income (low level as reference)**				
Middle level	-0.25*	0.00	0.00	-0.03
	(0.15)	(0.01)	(0.03)	(0.03)
High level	-0.08	-0.02	0.00	-0.01
	(0.14)	(0.01)	(0.03)	(0.03)
**Occupation (the unemployed as reference)**				
Manual workers	0.08	0.00	-0.03	0.02
	(0.17)	(0.02)	(0.03)	(0.04)
Agricultural workers	0.14	0.01	0.05	0.13***
	(0.19)	(0.01)	(0.04)	(0.03)
Self-employed	0.10	-0.03	0.01	0.13**
	(0.23)	(0.02)	(0.05)	(0.05)
Managers and professionals	0.32	-0.03	0.09**	0.17***
	(0.22)	(0.02)	(0.05)	(0.05)
**Education (no formal education as reference)**				
No formal education but can read/write	0.19	0.00	-0.02	0.05*
	(0.19)	(0.01)	(0.04)	(0.03)
Elementary school	0.07	-0.01	0.00	-0.06**
	(0.19)	(0.01)	(0.04)	(0.03)
Primary school	0.32	-0.02	-0.04	0.01
	(0.24)	(0.02)	(0.04)	(0.03)
High school and above	0.07	-0.05**	-0.23***	-0.02
	(0.29)	(0.02)	(0.04)	(0.06)
N	8,389	9,106	7,042	10,453

Note: The Sample Selection Model was used for male, and OLS was applied for female, urban and rural respondents. Demographic characteristics were controlled.

Significance level:

*** p<0.01,

** p<0.05,

* p<0.10.

Table 4 presents the associations between SES and high health risks related to smoking by Logit models among the middle aged and elderly Chinese population. Income was not related to health risks. Compared to the unemployed, all employed groups had significantly higher risks. For example, higher risks were found for managers and professionals among males, urban and rural residents by 91%, 82% and 90%, respectively. Compared to those with no formal education, lower risks were observed for those with high school education and above among males, females, and urban respondents by 39%, 77%, 55%, respectively.

**Table 4:** Association between SES and high health risk related to smoking using Logit model: OR with 95% CI.

	Male	Female	Urban	Rural
**Income (low level as reference)**				
Middle level	0.89	0.86	0.91	0.88
	(0.76–1.04)	(0.50–1.45)	(0.71–1.17)	(0.73–1.07)
High level	0.95	0.94	0.91	0.97
	(0.82–1.10)	(0.55–1.59)	(0.74–1.12)	(0.79–1.19)
**Occupation (the unemployed as reference)**				
Manual workers	1.30**	1.27	1.20	1.41**
	(1.03–1.65)	(0.71–2.27)	(0.87–1.67)	(1.03–1.93)
Agricultural workers	1.72***	1.57	1.49**	1.76***
	(1.37–2.15)	(0.88–2.83)	(1.03–2.17)	(1.36–2.28)
Self-employed	1.85***	0.73	1.87***	1.69***
	(1.39–2.46)	(0.19–2.74)	(1.23–2.84)	(1.19–2.40)
Managers and professionals	1.91***	1.09	1.82***	1.90***
	(1.48–2.46)	(0.40–2.98)	(1.26–2.62)	(1.39–2.60)
**Education (no formal education as reference)**				
No formal education but can read/write	0.98	1.06	0.83	1.05
	(0.78–1.22)	(0.61–1.84)	(0.56–1.23)	(0.82–1.35)
Elementary school	0.93	1.05	1.02	0.87
	(0.75–1.16)	(0.56–1.95)	(0.70–1.48)	(0.68–1.11)
Primary school	0.85	0.81	0.82	0.85
	(0.68–1.06)	(0.38–1.71)	(0.57–1.20)	(0.66–1.10)
High school and above	0.61***	0.23*	0.45***	0.87
	(0.47–0.79)	(0.05–1.10)	(0.30–0.68)	(0.62–1.23)
N	6,194	744	2,635	4,303

Note: Demographic characteristics were controlled.

Significance level:

*** p<0.01,

** p<0.05,

* p<0.10.

OR: odd ratio. CI: confidence interval.

Table 5 presents the associations between SES and HSI by Logit models among the middle aged and elderly Chinese population. Although agricultural workers were more likely to report higher HSI, there was no significant evidence supporting a relationship between smoking addiction and having a higher income, being a manager or other working professional. Individuals with higher educational attainment were less likely to experience smoking dependence. Compared to those with no formal education, smokers with high school education and above were less likely to be addicted to smoking by 31%, 65%, 36% and 27% among males, females, urban residents and rural residents, respectively.

**Table 5:** Association between SES and Heaviness of smoking index using ordered Logit model: OR with 95% CI.

	Male	Female	Urban	Rural
**Income (low level as reference)**				
Middle level	1.01	1.00	1.18	0.92
	(0.89–1.14)	(0.71–1.41)	(0.96–1.44)	(0.79–1.06)
High level	0.96	0.83	1.00	0.90
	(0.86–1.09)	(0.59–1.17)	(0.84–1.18)	(0.77–1.05)
**Occupation (the unemployed as reference)**				
Manual workers	1.01	1.17	0.98	1.16
	(0.83–1.22)	(0.81–1.71)	(0.76–1.27)	(0.91–1.47)
Agricultural workers	1.36***	1.46*	1.46**	1.40***
	(1.13–1.63)	(1.00–2.13)	(1.08–1.98)	(1.15–1.71)
Self-employed	1.15	0.63	1.12	1.11
	(0.91–1.45)	(0.31–1.30)	(0.81–1.57)	(0.84–1.48)
Managers and professionals	1.00	0.77	0.96	1.05
	(0.82–1.22)	(0.41–1.44)	(0.72–1.29)	(0.84–1.32)
**Education (no formal education as reference)**				
No formal education but can read/write	0.94	1.01	0.98	0.95
	(0.79–1.12)	(0.69–1.47)	(0.71–1.34)	(0.79–1.14)
Elementary school	0.82**	0.91	0.95	0.80**
	(0.69–0.97)	(0.62–1.33)	(0.71–1.27)	(0.67–0.96)
Primary school	0.73***	0.72	0.87	0.68***
	(0.62–0.87)	(0.41–1.26)	(0.65–1.18)	(0.56–0.83)
High school and above	0.59***	0.35***	0.54***	0.73**
	(0.48–0.72)	(0.16–0.75)	(0.39–0.75)	(0.57–0.95)
N	6,194	744	2,635	4,303

Note: The Heaviness of Smoking Index was classified into three ordered categories: 0, 1–3, and 4–6. Demographic characteristics were controlled.

Significance level:

*** p<0.01,

** p<0.05,

* p<0.10.

OR: odd ratio. CI: confidence interval.

## Discussion

Using nationally representative data in China, our study estimated the association between SES and smoking behaviors measured by smoking status, cigarette consumption, health risks, and smoking dependence among the middle aged and Elderly Chinese population. Although discrepancies were observed in the relationship between multiple measures of SES and smoking behaviors, in general, China fit characteristics in the third or fourth stages of the smoking epidemic model. Our results revealed a small or insignificant relationship between income and smoking behaviors, and a significant gradient in multiple measures of smoking behaviors by occupation and education.

First, although managers and professionals were more likely to smoke than unemployed respondents in China, they were not more likely to develop smoking dependence. This association may be related to Chinese socio-cultural characteristics.[53] In Chinese society, cigarettes are generally considered as popular gifts, and the gifting and exchanging of cigarettes appears to be very common [54,55]. Gifting cigarettes may help build interpersonal relationships through a number of daily interactions and social occasions [2,3]. The gifting of expensive premium cigarettes may play an important role in displaying social position, and facilitating business and government affairs [46]. Therefore, individuals with occupations such as managers and professionals appear particularly prone to the cigarette-gifting norm [56]. Since managers and professionals may only consider cigarettes as a means of social communication, they may be at less risk of developing smoking dependence.

Second, income was found to be small or even insignificantly related to smoking behaviors in China. Studies from high-income countries revealed that the independent effect of income was less pronounced or absent, but indeed occurred in low-income countries and some middle-income countries [57–59]. A possible explanation is that cigarette consumption only accounts for a small share of an individual’s disposable income in high-income countries but a large share of income in low-income countries. Therefore, in high-income countries, budget concerns may not force individuals to reduce cigarette consumption or quit smoking. In China, household income has grown considerably faster than expenditure on cigarettes in recent years. From 1990 to 2005, the growth of household income was nearly three times greater than cigarette expenditure, and the ability to pay for cigarettes has doubled [60]. In addition, high-income individuals are more prone to increased work-related stress, which could be overbearing. As a result, there is a chance that high-income individuals may cope with job-related stress by smoking [61]. Job stress may cushion the potential favorable effects of income on smoking. These findings may explain why income had little influence in smoking behaviors in China.

Third, in accordance with the findings from high-income countries, our results indicate that individuals with less education exhibit a greater degree of smoking behaviors. The association of education and smoking behaviors may be mediated by health knowledge. It is well known that education is a robust determinant of health awareness [62]. Exposure to health education improves understanding of the association between health behaviors and outcomes, positively contributing to healthy behaviors [63]. There is plenty of evidence supporting the viewpoint that higher educational levels and better health knowledge can promote healthy behaviors [64]. Furthermore, less educated individuals may be less responsive to health promotion, receive less information about the consequences of smoking, and have limited access to cessation services [65].

Our findings also indicated that in China, education displayed a stronger relationship with smoking than income. Similar relationships were also found in European Union data [19]. Huisman et al. provided several explanations for this phenomenon. Education, instead of income, provides individuals with more health knowledge, and prevents them from coping with adverse events related to smoking [19]. In addition, because school performance is associated with smoking initiation and socioeconomic status during early life, it is a decisive period for future smoking status. In general, educational levels were found to be an indicator of future smoking status [66, 67]. Thus, education can reflect these effects more accurately than income.

Furthermore, smoking patterns in SES differed by urban and rural residence. No significant difference in cigarette consumption and smoking-related risks was observed between respondents with high school education and above or those with no formal education in rural areas. Conversely, urban residents with high school education and above were likely to have lower cigarette consumption and health risks related to smoking. These differential smoking patterns in SES may be due to the educational background among rural or urban residents. Compared to urban residents, educational attainment was significantly lower in rural areas. Additionally, 17% of middle aged and elderly urban respondents had a high school education or above, whereas only 5% of rural counterparts had a similar education level. As a result of the small proportion in rural areas, respondents with a high school education or above were more likely to be exposed to a smoking environment and obtain an insignificant difference in cigarette consumption by education.

The comparison of smoking patterns in SES by gender were more complex. We found that males were more likely to smoke and have health risks if they had a higher income or worked as a manager or other professional. Conversely, their female counterparts were observed to be insignificantly or negatively associated with smoking and related health risks. In addition, females with high school education or above smoked fewer cigarettes than other groups, however this association was not found for males. The Chinese socio-cultural characteristics might be a major factor in the large discrepancy between males and females. Although the Chinese society has been influenced by the western culture during past decades, it remains quite conservative and rooted into the traditional Confucian culture. The Chinese society is quite tolerant to male smoking but not female smoking [68]. Females with higher SES may be more sensitive to social norms against smoking, and more likely to be exposed to smoking restrictions and social pressures at their workplace [69,70].

Overall, our findings highlight the comprehensive approaches to smoking reduction and cessation. First, to reduce the smoking prevalence among managers and professionals, social culture and attitudes toward gifting cigarettes should be improved. Policies that make cigarette packaging less attractive would probably reduce the value of cigarettes for gifting. Second, due to the negative association between individual education and smoking behaviors, tobacco control policies should focus on populations with a low level of education in China. To break the link between educational disadvantage and smoking status, tobacco control policies should pay more attention on enhancing health literacy and anti-smoking education initiatives. Third, the tailored interventions should target smoking characteristics of different SES groups. The tailored interventions may include the improvement of awareness regarding social norms of tobacco control for low-income females, and offering social support for rural residents. Therefore, the tobacco control policies should not only address individual behaviors, but also mitigate broader inequalities in educational opportunities and cultural backgrounds [71].

### Contributions and limitations

The study makes three major contributions to the existing literature. Primarily, this is the first study that evaluates the effects of SES on smoking behaviors in the under-studied middle aged and elderly Chinese population using nationally representative data. Most evidence about the effects of SES on smoking has been derived from data reported in developed countries. Given the low levels of tobacco consumption in developed countries, the estimations may not be valid in developing countries. On the other hand, the macroeconomic environment and education levels of the population in China are drastically different from those of developed countries. Thus, investigating these relationships in China and comparisons to the developed countries may be insightful.

Second, different indicators of SES and smoking behaviors were included in the study.^8^ The current literature generally estimated only single measures of SES or smoking behaviors, such as smoking prevalence rates by educational level. This study evaluated the effects of SES (income, occupation and education) on various measures of smoking, including smoking dependence. A comprehensive understanding of the socioeconomic determinants of smoking behaviors would support the tailored interventions by SES to reduce unhealthy smoking habits.

Third, cigarette consumption, health risks, and smoking dependence may be a non-random sample of all respondents if SES variables affect the decision to smoke. As a result, the OLS or Logit model may produce biased results in the sample of smoking accumulation. In our study, the SSM model was used to adjust for the selection bias.

The interpretation of our results is subject to the current study’s limitations. First, the evidence that relates SES to smoking behaviors is restricted in the cross-sectional study and among Chinese aged 45 and above. A Longitudinal study aiming to examine trends in the association between SES and smoking behaviors targeting whole Chinese population would be more valuable, and would accurately determine the stage in the smoking epidemic model. Second, qualitative research is recommended to understand the reasons for smoking patterns by SES. Our study showed that reducing the current high smoking prevalence among men with low SES is an important public health goal in China. Achieving this goal will require more knowledge regarding how SES influences smoking behaviors.

## Conclusions

Our study adds to the growing body of literature on the relationship between SES and smoking behaviors, and found discrepancies in the relationship between multiple measures of SES and smoking behaviors. Although we detected a small or insignificant relationship between income and smoking behaviors, a significant gradient in multiple measures of smoking behaviors by occupation and education were observed among the middle aged and elderly Chinese populaiton. Individuals with less education exhibit a greater degree of smoking behaviors. Managers and professionals were more likely to smoke than the unemployed, but there was no significant relationship with smoking dependence. Furthermore, gender and urban-rural differences were found in the relationship between SES and smoking behaviors. Accordingly, our study suggests China’s tobacco control policies should mitigate inequalities in education, improve the social culture of cigarettes, and tailor interventions based on the characteristics of the population.

## References

[1] Eriksen M, Mackay J, Ross H. The Tobacco Atlas, 4^th^ ed New York: World Lung Foundation, 2012.

[2] World Health Organization. WHO Report on the global tobacco epidemic 2008. Geneva: World Health Organization, 2008.

[3] Lantz PM, House JS, Lepkowski JM, Williams DR, Mero RP, Chen J. Socioeconomic factors, lifestyles, and mortality: results from a nationally representative prospective study of US adults. *JAMA*. 1998; 279: 1703–1708. 9624022 10.1001/jama.279.21.1703

[4] Rogers EM. Diffusion of Innovations. 4th ed New York, NY: Free Press, 1995.

[5] Lopez AD. The lung cancer epidemic in developed countries In: Lopez AD, Caselli G, Valkonen T, eds. Adult Mortality in Developed Countries: From Description to Explanation. Oxford, United Kingdom: Clarendon Press, 1995, pp 111–143.

[6] Doku D, Koivusilta L, Rainio S, Rimpelä A. Socioeconomic differences in smoking among Finnish adolescents from 1977 to 2007. *Journal of Adolescent Health*. 2010; 47(5): 479–487. doi: 10.1016/j.jadohealth.2010.03.012 20970083 10.1016/j.jadohealth.2010.03.012

[7] Schaap MM, Kunst AE. Monitoring of socio-economic inequalities in smoking: learning from the experiences of recent scientific studies. *Public Health*. 2009; 123(2): 103–109. doi: 10.1016/j.puhe.2008.10.015 19147163 10.1016/j.puhe.2008.10.015

[8] Hanibuchi T, Nakaya T, Honjo K. Trends in socioeconomic inequalities in self-rated health, smoking, and physical activity of Japanese adults from 2000 to 2010. *SSM- Population Health*. 2016; 2: 662–673. doi: 10.1016/j.ssmph.2016.09.002 29349178 10.1016/j.ssmph.2016.09.002 PMC5757786

[9] Cavelaars AE, Kunst AE, Geurts JJ, Crialesi R, Grötvedt L, Helmert U, et al Educational differences in smoking: international comparison. *BMJ*. 2000;320:1102–1107. 10775217 10.1136/bmj.320.7242.1102 PMC27351

[10] Song Y-M, Byeon JJ. Excess mortality from avoidable and non-avoidable causes in men of low socioeconomic status: a prospective study in Korea. *Journal of Epidemiology Community Health*. 2000; 54:166–172. doi: 10.1136/jech.54.3.166 10746109 10.1136/jech.54.3.166 PMC1731641

[11] Cho HJ, Song YM, Smith GD, Ebrahim S. Trends in socio-economic differentials in cigarette smoking behaviour between 1990 and 1998: a large prospective study in Korean men. *Public Health*. 2004; 118(8): 553–558. doi: 10.1016/j.puhe.2004.04.006 15530934 10.1016/j.puhe.2004.04.006

[12] Laaksonen M, Rahkonen O, Karvonen S, Lahelma E. Socioeconomic status and smoking: analysing inequalities with multiple indicators. *European Journal of Public Health*. 2005; 15(3): 262 doi: 10.1093/eurpub/cki115 15755781 10.1093/eurpub/cki115

[13] Lemstra M, Mackenbach J, Neudorf C, Nannapaneni U, Kunst A. Daily smoking in Saskatoon: the independent effect of income and cultural status. Canadian journal of public health, 2009; 100(1):51 19263984 10.1007/BF03405493 PMC6973952

[14] Bosdriesz JR, Mehmedovic S, Witvliet MI, Kunst AE. Socioeconomic inequalities in smoking in low and mid income countries: positive gradients among women? *International journal for equity in health*. 2014; 13(1):14. 24502335 10.1186/1475-9276-13-14 PMC3922442

[15] Hosseinpoor AR, Parker LA, D’Espaignet ET, Chatterji S. Socioeconomic Inequality in Smoking in Low-Income and Middle-Income Countries: Results from the World Health Survey. *Plos One*. 2012; 7(8): e42843 doi: 10.1371/journal.pone.0042843 22952617 10.1371/journal.pone.0042843 PMC3430638

[16] Siahpush M, Borland R, Yong HH, Kin F, Sirirassamee B. Socio-economic variations in tobacco consumption, intention to quit and self-efficacy to quit among male smokers in Thailand and Malaysia: results from the international tobacco control–south-east Asia (itc–sea) survey. *Addiction*. 2008; 103(3): 502–508. doi: 10.1111/j.1360-0443.2007.02113.x 18269370 10.1111/j.1360-0443.2007.02113.x

[17] Schaap MM, Kunst AE, Mall L, Regidor E, Ekholm O, Helmert U, et al Female ever-smoking, education, emancipation and economic development in 19 European countries. *Social Science & Medicine*. 2009; 68(7): 1271–1278. 19195749 10.1016/j.socscimed.2009.01.007

[18] Legleye S, Khlat M, Beck F, Peretti-Watel P. Widening inequalities in smoking initiation and cessation patterns: a cohort and gender analysis in France. *Drug & Alcohol Dependence*. 2011; 117(2–3): 233–241. 21420251 10.1016/j.drugalcdep.2011.02.004

[19] Huisman M, Kunst AE, Mackenbach JP. Inequalities in the prevalence of smoking in the European union: comparing education and income. *Preventive Medicine*. 2005; 40(6): 756–764. doi: 10.1016/j.ypmed.2004.09.022 15850876 10.1016/j.ypmed.2004.09.022

[20] Swe Latt, Razman MR, Jamalludin AR, Jamalludin N, Hashima E, Phyu, HM, et al. Nicotine dependency of adult male smokers and it’s socio-economic determinants. In: 18th Family Medicine Scientific Conference 2015, Kuala Terenganu.

[21] Siahpush M, McNeill A, Borland R, Fong GT. Socioeconomic variations in nicotine dependence, self-efficacy, and intention to quit across four countries: findings from the International Tobacco Control (ITC) Four Country Survey. *Tobacco Control*. 2006; 15 Suppl 3:iii71–5. 16754950 10.1136/tc.2004.008763 PMC2593052

[22] Villanti AC, Johnson AL, Rath JM. Beyond education and income: Identifying novel socioeconomic correlates of cigarette use in U.S. young adults. *Preventive Medicine*. 2017; 104: 63–70. doi: 10.1016/j.ypmed.2017.06.019 28647547 10.1016/j.ypmed.2017.06.019 PMC5857885

[23] Barik A, Rai RK, Gorain A, Majumdar S, Chowdhury A. Socio-economic disparities in tobacco consumption in rural india: evidence from a health and demographic surveillance system. *Perspectives in Public Health*. 2015; 136(5): 1–5. 10.1177/1757913915609947 26475772

[24] Link B G, Phelan J, Higgins S T, Chilcoat HD. The social shaping of health and smoking. *Drug Alcohol Depend*. 2009; 104(3):S6–S10. 19477607 10.1016/j.drugalcdep.2009.03.002

[25] Federico B, Kunst AE, Vannoni F, Damiani G, Costa G. Trends in educational inequalities in smoking in northern, mid and southern Italy, 1980–2000. *Preventive Medicine*. 2004; 39(5): 919–926. doi: 10.1016/j.ypmed.2004.03.029 15475024 10.1016/j.ypmed.2004.03.029

[26] Chinese Center for Disease Control and Prevention. Global Adult Tobacco Survey (GATS) China Country Report 2010. Beijing: China Sanxia Press, 2011.

[27] Chinese Center for Disease Control and Prevention. China Adult Tobacco Survey Report 2015. http://www.ce.cn/xwzx/gnsz/gdxw/201512/28/t20151228_7911737.shtml. Accessed November 28, 2015.

[28] Lim SS, Vos T, Flaxman AD, Danaei G, Shibuya K, Adair-Rohani H, et al A comparative risk assessment of burden of disease and injury attributable to 67 risk factors and risk factor clusters in 21 regions, 1990–2010: A systematic analysis for the global burden of disease study 2010. *Lancet*. 2013; 380: 2224–2260. 10.1016/S0140-6736(12)61766-8 PMC4156511 23245609

[29] Le C, Xinan W, Abhinav G, Han Y, Cui W, Xiao X, et al Patterns and socioeconomic influences of tobacco exposure in tobacco cultivating rural areas of Yunnan province, China. *BMC Public Health*. 2012; 12(1): 1–8. 23035644 10.1186/1471-2458-12-842 PMC3515419

[30] Le C., Cui W., You D., He J., Zhao K. Socioeconomic variations in nicotine dependence in rural southwest china. *BMC Public Health*. 2015; 15(1): 1–6. 26597724 10.1186/s12889-015-2492-9 PMC4657195

[31] Yang T, Shiffman S, Rockett IR, Cui X, Cao R. Nicotine dependence among Chinese city dwellers: a population-based cross-sectional study. *Nicotine Tobacco Research*. 2011;13:556–564. doi: 10.1093/ntr/ntr040 21454911 10.1093/ntr/ntr040

[32] Yong HH, Siahpush M, Borland R, Li L, O’Connor RJ, Yang J, et al Urban Chinese Smokers From Lower Socioeconomic Backgrounds Face More Barriers to Quitting: Results From the International Tobacco Control-China Survey. *Nicotine Tobacco Research*. 2013;15:1044–51. doi: 10.1093/ntr/nts234 23125438 10.1093/ntr/nts234 PMC3646648

[33] Gruder CL, Trinidad DR, Palmer PH, Xie B, Li L, Anderson C, et al Tobacco smoking, quitting, and relapsing among adult males in Mainland China: the China Seven Cities Study. *Nicotine Tobacco Research*. 2013; 15: 223–230. doi: 10.1093/ntr/nts116 22581939 10.1093/ntr/nts116 PMC3611989

[34] Yang T, Li F, Yang X, Xu Z, Feng X, Wang X, et al Smoking patterns and sociodemographic factors associated with tobacco use among Chinese rural male residents: a descriptive analysis. *BMC Public Health*. 2008; 8(1): 248. 18644139 10.1186/1471-2458-8-248 PMC2492861

[35] Zhang D, Shi L, Tian F, Zhang L. Care utilization with China’s New Rural Cooperative Medical Scheme: Updated evidence from the China Health and Retirement Longitudinal Study 2011–2012. *International Journal of Behavioral Medicine*. 2016; 23(6): 655–663. doi: 10.1007/s12529-016-9560-0 27102433 10.1007/s12529-016-9560-0

[36] Li Q, Hsia J, Yang G. Prevalence of Smoking in China in 2010. *New England Journal of Medicine*. 2011; 364(25):2469 doi: 10.1056/NEJMc1102459 21696322 10.1056/NEJMc1102459

[37] Wang Q, Shen JJ, Cochran C. Unemployment rate, smoking in china: are they related?. *International Journal of Environmental Research & Public Health*, 2016; 13(1). 10.3390/ijerph13010113 PMC4730504 26761019

[38] Leffondré K, Abrahamowicz M, Xiao Y, Siemiatycki J, Modelling smoking history using a comprehensive smoking index: application to lung cancer. *Statistics in Medicine*. 2006; 25(24): 4132–4146. doi: 10.1002/sim.2680 16998807 10.1002/sim.2680

[39] Roberts B, Gilmore A, Stickley A, Kizilova K, Prohoda V, Rotman D, et al Prevalence and psychosocial determinants of nicotine dependence in nine countries of the former Soviet Union. *Nicotine Tobacco Research*. 2013; 15:271–6. doi: 10.1093/ntr/nts100 22529221 10.1093/ntr/nts100

[40] Manimunda SP, Benegal V, Sugunan AP, Jeemon P, Balakrishna N, Thennarusu K, et al Tobacco use and nicotine dependency in a cross- sectional representative sample of 18,018 individuals in Andaman and Nicobar Islands, India. *BMC Public Health*. 2012; 12: 515 doi: 10.1186/1471-2458-12-515 22781062 10.1186/1471-2458-12-515 PMC3439319

[41] Pennanen M, Broms U, Korhonen T, Haukkala A, Partonen T, Tuulio-Henriksson A, et al Smoking, nicotine dependence and nicotine intake by socio-economic status and marital status. *Addictive Behaviors*. 2014; 39(7): 1145–1151. doi: 10.1016/j.addbeh.2014.03.005 24727110 10.1016/j.addbeh.2014.03.005

[42] Silagy C, Lancaster T, Stead L, Partonen T, Tuulio-Henriksson A, Laatikainen T, et al Nicotine replacement therapy for smoking cessation. *Cochrane database system review*. 2004; (3):cd000146. 10.1002/14651858.CD000146.pub2 15266423

[43] Haddock CK, Lando H, Klesges RC, Talcott GW, Renaud EA. A study of the psychometric and predictive properties of the Fagerström Test for Nicotine Dependence in a population of young smokers. *Nicotine & Tobacco Research*. 1999; 1(1): 59–66. 11072389 10.1080/14622299050011161

[44] Heatherton TF, Kozlowski LT, Frecker RC, Fagerström KO. The Fagerström Test for Nicotine Dependence: A revision of the Fagerström Tolerance Questionnaire. *British Journal of Addiction*. 1991; 86(9): 1119–1127. 1932883 10.1111/j.1360-0443.1991.tb01879.x

[45] Patrick DL, Cheadle A, Thompson DC, Diehr P, Koepsell T, Kinne S. The validity of self-reported smoking and meta-analysis. *American Journal of Public Health*. 1994; 84(7): 1086–1093. 8017530 10.2105/ajph.84.7.1086 PMC1614767

[46] Vartiainen E, Seppälä T, Lillsunde P, Puska P. Validation of self-reported smoking by serum cotinine measurement in a community-based study. *Journal of Epidemiology and Community Health*. 2002; 56(3): 167–170. doi: 10.1136/jech.56.3.167 11854334 10.1136/jech.56.3.167 PMC1732104

[47] Hu TW, Mao Z, Shi J, Chen W. Tobacco Taxation and Its Potential Impact in China. Paris: International Union Against Tuberculosis and Lung Disease, 2008.

[48] Datta G, Meerman J. household income or household income per capita in welfare comparisons. *Review of Income & Wealth*. 1980; 26(4):401–418.

[49] Xie Z, Poon A N, Wu Z, Jian W, Chan KY. Is Occupation a Good Predictor of Self-Rated Health in China? *Plos One*. 2015, 10(5):e0125274 doi: 10.1371/journal.pone.0125274 25951087 10.1371/journal.pone.0125274 PMC4423882

[50] Chen M, Wu Y, Narimatsu H, Li X, Wang C, Luo J, et al Socioeconomic status and physical activity in Chinese adults: a report from a community-based survey in Jiaxing, China. *Plos One*. 2015, 10(7): e0132918 doi: 10.1371/journal.pone.0132918 26177205 10.1371/journal.pone.0132918 PMC4503452

[51] Greenlund KJ, Liu K, Kiefe CI, Yunis C, Dyer AR, Burke GL. Impact of Father’s Education and Parental Smoking Status on Smoking Behavior in Young Adults: The CARDIA Study. *American Journal of Epidemiology*. 1995; 142(10):1029–1033. 7485047 10.1093/oxfordjournals.aje.a117555

[52] Heckman J. The common structure of statistical models of truncation, sample selection and limited dependent variables and a simple estimator for such models. *Annals of Economic and Social Measurement*. 1974; 5: 475–492.

[53] Kendzor DE, Reitzel LR, Mazas CA, Cofta-woerpel LM, Cao Y, Ji L, et al Individual- and area-level unemployment influence smoking cessation among African Americans participating in a randomized clinical trial. *Social Science and Medicine*. 2012; 74(9): 1394–1401. doi: 10.1016/j.socscimed.2012.01.013 22405506 10.1016/j.socscimed.2012.01.013 PMC3321106

[54] Chu A, Jiang N, Glantz SA. Transnational tobacco industry promotion of the cigarette gifting custom in China. *Tobacco Control*. 2011; 20:e3. 10.1136/tc.2010.038349 PMC3124594 21282136

[55] Rich ZC, Xiao S. Tobacco as a social currency: cigarette gifting and sharing in China. *Nicotine Tobacco Research*. 2012; 14: 258–263. doi: 10.1093/ntr/ntr156 21849412 10.1093/ntr/ntr156

[56] Huang LL, Thrasher JF, Yuan J, Li Q, Fong GT, Quah AC, et al Incidence and correlates of receiving cigarettes as gifts and selecting preferred brand because it was gifted: findings from the itc China survey. *BMC Public Health*. 2012; 12(26): 3241–3252. 10.1186/1471-2458-12-996 PMC3532818 23157697

[57] Hosseinpoor AR, Parker LA, D’Espaignet ET, Chatterji S. Social Determinants of Smoking in Low- and Middle-Income Countries: Results from the World Health Survey. *Plos One*. 2011; 6(5):e20331 doi: 10.1371/journal.pone.0020331 21655299 10.1371/journal.pone.0020331 PMC3105024

[58] Huisman M, Kunst AE, Mackenbach JP. Inequalities in the prevalence of smoking in the European Union: comparing education and income. *Preventive Medicine*. 2005; 40(6):756 doi: 10.1016/j.ypmed.2004.09.022 15850876 10.1016/j.ypmed.2004.09.022

[59] Bosdriesz JR, Mehmedovic S, Witvliet MI, Kunst AE. Socioeconomic inequalities in smoking in low and mid income countries: positive gradients among women? *International journal for equity in health*. 2014; 13(1):14. 24502335 10.1186/1475-9276-13-14 PMC3922442

[60] Falba T, Teng HM, Sindelar JL, Gallo WT. The effect of involuntary job loss on smoking intensity and relapse. *Addiction*. 2005; 100: 1330–1339. doi: 10.1111/j.1360-0443.2005.01150.x 16128722 10.1111/j.1360-0443.2005.01150.x PMC1351253

[61] Ruhm CJ. Good times make you sick. *Journal of Health Economics*. 2003; 22(4): 637 doi: 10.1016/S0167-6296(03)00041-9 12842319 10.1016/S0167-6296(03)00041-9

[62] Glanz K, Rimer BK, Viswanath K. *Health behavior and health education*: *theory*, *research*, *and practice*. New York: John Wiley and Sons, 2008.

[63] Grossman M. The demand for health: a theoretical and empirical investigation. New York: Columbia Univ. Press, 1971.

[64] Grossman M. The correlation between health and schooling In: Nestor E. Terleckyj, Editors. Household Production and Consumption. New York: Columbia University Press,1976, pp. 147–247.

[65] Berger MC, Leigh JP. Schooling, self-Selection, and health. *The Journal of Human Resources*.1989; 24: 433–455.

[66] Simons-Morton B, Haynie DL, Crump AD, Eitel P, Saylor KE. Peer and parent influences on smoking and drinking among early adolescents. *Health Educational Behavior*. 2001; 28: 95–107. 10.1177/109019810102800109 11213145

[67] Gilman S, Abrams D, Buka S. Socioeconomic status over the life course and stages of cigarette use: initiation, regular use, and cessation. *Journal of Epidemiology Community Health*. 2003; 57:802–8. doi: 10.1136/jech.57.10.802 14573586 10.1136/jech.57.10.802 PMC1732304

[68] Li S, Meng L, Chiolero A, Ma C, Xi B. Trends in smoking prevalence and attributable mortality in China, 1991–2011. *Preventive Medicine*. 2016; 93:82–87. doi: 10.1016/j.ypmed.2016.09.027 27677441 10.1016/j.ypmed.2016.09.027 PMC5124560

[69] Bauer T, Göhlmann S, Sinning M. Gender differences in smoking behavior. *Health Economics*. 2007; 16(9):895–909. doi: 10.1002/hec.1259 17619229 10.1002/hec.1259

[70] Liu S, Mei Z, Ling Y, Li Y, Wang L, Huang Z, et al Prevalence and patterns of tobacco smoking among Chinese adult men and women: findings of the 2010 national smoking survey. *Journal of Epidemiology & Community Health*. 2016; 71(2):154–161. 27660401 10.1136/jech-2016-207805 PMC5284482

[71] Hosseinpoor AR, Parker LA, D’Espaignet ET, Chatterji S. Social Determinants of Smoking in Low- and Middle-Income Countries: Results from the World Health Survey. *Plos One*. 2011; 6(5):e20331 doi: 10.1371/journal.pone.0020331 21655299 10.1371/journal.pone.0020331 PMC3105024

